# Evolutionary genetic algorithm identifies *IL2RB* as a potential predictive biomarker for immune-checkpoint therapy in colorectal cancer

**DOI:** 10.1093/nargab/lqab016

**Published:** 2021-04-20

**Authors:** Matthew Alderdice, Stephanie G Craig, Matthew P Humphries, Alan Gilmore, Nicole Johnston, Victoria Bingham, Vicky Coyle, Seedevi Senevirathne, Daniel B Longley, Maurice B Loughrey, Stephen McQuaid, Jacqueline A James, Manuel Salto-Tellez, Mark Lawler, Darragh G McArt

**Affiliations:** Patrick G Johnston Centre for Cancer Research, Queen’s University Belfast, BT9 7AE, Northern Ireland; Health Data Research UK Wales and Northern Ireland; Precision Medicine Centre of Excellence, Patrick G Johnston Centre for Cancer Research, Queen’s University Belfast, Belfast, BT9 7AE, Northern Ireland; Precision Medicine Centre of Excellence, Patrick G Johnston Centre for Cancer Research, Queen’s University Belfast, Belfast, BT9 7AE, Northern Ireland; Patrick G Johnston Centre for Cancer Research, Queen’s University Belfast, BT9 7AE, Northern Ireland; Patrick G Johnston Centre for Cancer Research, Queen’s University Belfast, BT9 7AE, Northern Ireland; Patrick G Johnston Centre for Cancer Research, Queen’s University Belfast, BT9 7AE, Northern Ireland; Precision Medicine Centre of Excellence, Patrick G Johnston Centre for Cancer Research, Queen’s University Belfast, Belfast, BT9 7AE, Northern Ireland; Patrick G Johnston Centre for Cancer Research, Queen’s University Belfast, BT9 7AE, Northern Ireland; Patrick G Johnston Centre for Cancer Research, Queen’s University Belfast, BT9 7AE, Northern Ireland; Patrick G Johnston Centre for Cancer Research, Queen’s University Belfast, BT9 7AE, Northern Ireland; Patrick G Johnston Centre for Cancer Research, Queen’s University Belfast, BT9 7AE, Northern Ireland; Patrick G Johnston Centre for Cancer Research, Queen’s University Belfast, BT9 7AE, Northern Ireland; Precision Medicine Centre of Excellence, Patrick G Johnston Centre for Cancer Research, Queen’s University Belfast, Belfast, BT9 7AE, Northern Ireland; Precision Medicine Centre of Excellence, Patrick G Johnston Centre for Cancer Research, Queen’s University Belfast, Belfast, BT9 7AE, Northern Ireland; Precision Medicine Centre of Excellence, Patrick G Johnston Centre for Cancer Research, Queen’s University Belfast, Belfast, BT9 7AE, Northern Ireland; Patrick G Johnston Centre for Cancer Research, Queen’s University Belfast, BT9 7AE, Northern Ireland; Health Data Research UK Wales and Northern Ireland; Patrick G Johnston Centre for Cancer Research, Queen’s University Belfast, BT9 7AE, Northern Ireland; Health Data Research UK Wales and Northern Ireland; Precision Medicine Centre of Excellence, Patrick G Johnston Centre for Cancer Research, Queen’s University Belfast, Belfast, BT9 7AE, Northern Ireland

## Abstract

Identifying robust predictive biomarkers to stratify colorectal cancer (CRC) patients based on their response to immune-checkpoint therapy is an area of unmet clinical need. Our evolutionary algorithm Atlas Correlation Explorer (ACE) represents a novel approach for mining The Cancer Genome Atlas (TCGA) data for clinically relevant associations. We deployed ACE to identify candidate predictive biomarkers of response to immune-checkpoint therapy in CRC. We interrogated the colon adenocarcinoma (COAD) gene expression data across nine immune-checkpoints (*PDL1, PDCD1, CTLA4, LAG3, TIM3, TIGIT, ICOS, IDO1* and *BTLA*). *IL2RB* was identified as the most common gene associated with immune-checkpoint genes in CRC. Using human/murine single-cell RNA-seq data, we demonstrated that *IL2RB* was expressed predominantly in a subset of T-cells associated with increased immune-checkpoint expression (*P* < 0.0001). Confirmatory IL2RB immunohistochemistry (IHC) analysis in a large MSI-H colon cancer tissue microarray (TMA; *n* = 115) revealed sensitive, specific staining of a subset of lymphocytes and a strong association with FOXP3+ lymphocytes (*P* < 0.0001). *IL2RB* mRNA positively correlated with three previously-published gene signatures of response to immune-checkpoint therapy (*P* < 0.0001). Our evolutionary algorithm has identified *IL2RB* to be extensively linked to immune-checkpoints in CRC; its expression should be investigated for clinical utility as a potential predictive biomarker for CRC patients receiving immune-checkpoint blockade.

## INTRODUCTION

Colorectal cancer (CRC) is one of the world’s leading causes of cancer-related mortality. Recent advances in our understanding of the immune landscape in CRC, coupled with the development of immune-checkpoint therapy has underpinned improved outcomes for a subset of deficient mismatch repair (dMMR) CRC patients (1,2). Immune-checkpoints regulate the host immune response by modulating activity of immune cells in the tumor microenvironment (TME), including CD8+ cytotoxic lymphocytes (CTLs) and natural killer (NK) cells. Dysregulation of immune-checkpoints results in immune-evasion, one of the major hallmarks of cancer. The discovery that targeting costimulatory and inhibitory immune-checkpoints can invoke a CTL/NK cell response against tumor cells has provided the rationale for a new immunotherapy-based treatment (3,4).

The first immune-checkpoint therapy to receive FDA-approval was Ipilimumab (anti-CTLA4) in 2011 for advanced melanoma (5). Since then, an evolving armamentarium of immune-checkpoint compounds have undergone preclinical and early clinical investigation across many cancer types including CRC (2,6). Despite rigorous research, to date only CTLA-4 and PD-1 inhibitors have been FDA-approved for the treatment of dMMR metastatic CRC (mCRC) previously treated with chemotherapy (7,8). Clinical indication for PD-1 inhibitors is currently limited to patients with dMMR and hypermutated tumors (e.g., microsatellite instability (MSI-H) and POLE mutations). PDL1 expression by immunohistochemistry (IHC) is employed for stratification in other tumor types such as non-small cell lung cancer (NSCLC); however, it is not routinely used as a predictive biomarker for CRC. Anti-CTLA4 (NCT03007407) in combination with PD-1 inhibition has reached phase II/III clinical trials, while other immune-checkpoint inhibitors including anti-LAG-3 (NCT 02060188), anti-TIM-3 (NCT02817633) and anti-IDO (NCT 02048709) are currently being trialled in combination or as single agents. However, there is a dearth of robust predictive biomarkers to inform immune-checkpoint approaches.

In the era of precision medicine, high-throughput molecular profiling of tumors to identify biomarkers for patient stratification is requiring more sophisticated computational analysis. Artificial intelligence (AI) approaches employing machine learning, neural networks and evolutionary genetic algorithms are starting to address this need in domains such as disease screening, molecular characterization and pathological image analysis (9–13). Recently, Ruiz-Bañobre and Goel highlighted how AI algorithms will be key in deciphering response to immune checkpoints in dMMR gastrointestinal tumors (14). We have previously published Atlas Correlation Explorer (ACE), which implements an evolutionary genetic algorithm that extracts associations from molecular data within The Cancer Genome Atlas (TCGA) to facilitate biomarker discovery (15). ACE eschews a linear and computationally intensive approach in favor of a genetic algorithm-based heuristic search method that rapidly generates succinct feature lists where clinical associations across analyses can be more easily determined.

In this study, we have employed ACE to assess common associations in gene expression across nine immune co-stimulatory/inhibitory checkpoints within the TCGA CRC cohort. We hypothesized that commonality of gene expression across immune checkpoints may allow selection of one or more overarching biomarkers of patient outcome and response to immune-checkpoint blockade in CRC. Our analysis identified Interleukin-2 receptor subunit beta (*IL2RB)* as the most common gene associated with immune-checkpoint gene expression in CRC. *IL2RB*, also known as CD122, has been shown to be associated with not only T-cell expansion, but also T-cell exhaustion (16–18); it is a promising therapeutic target under investigation in combination with immune-checkpoint blockade in phase II/III clinical trials for patients with advanced solid tumors (19). We have established that expression of *IL2RB* is associated with increased immune infiltrates and is prognostic at the mRNA level, further validating this finding in an independent cohort. We demonstrated using publically available human and murine single cell RNA-seq that *IL2RB* is expressed predominantly in a subset of T-cells which are associated with increased immune-checkpoint expression. We have optimized digital pathology analysis of IL2RB IHC to further demonstrate its specific expression on a population of tumor infiltrating lymphocytes (TILs). Finally, we demonstrated that *IL2RB* mRNA expression is positively correlated with predictive gene signatures for response to anti-PD1 and anti-PDL1 therapy. On this basis, we hypothesize that *IL2RB* expression may yield predictive value in prospective clinical trials for immune-checkpoint blockade therapy in CRC.

## MATERIALS AND METHODS

### Atlas Correlation Explorer (ACE) analysis and gene list intersections

ACE was installed as described in our previous publication (15). ACE is written in C# and Microsoft Visual Studio and implemented as a Windows desktop application. It can be accessed at GitHub (https://github.com/AlanRGilmore/ACE). ACE uses TCGA data directly from the Broad Institute Firehose https://gdac.broadinstitute.org/. Analysis of immune-checkpoints genes in the TCGA COAD dataset was performed using the Agilent microarray (median expression) (*n* = 153) and RNA-seq (uncv2.mRNAseq_RSEM_normalized_log2) (*n* = 457) pipelines. Each analysis was performed until 100% coverage was achieved and exported feature lists were filtered using a criteria of *R*^2^>0.25. The proportion of overlap/intersections between gene lists was assessed using Upset plots (20) implemented in R version 3.3.1 (2016–06–21) – ‘Bug in Your Hair’.

### Immune-checkpoint source measures

The following nine immune-checkpoint molecules were identified from the literature for analysis using ACE and are described in Table 1; exported gene lists were assessed for commonality in the CRC TCGA microarray and RNA-seq data.

**Table 1.** Table displays the gene name and description for each immune checkpoint biomarker analyzed by ACE

Immune checkpoint	Description	References
PDL1	Programmed death-ligand 1 (PD-L1), also known as CD274, is the ligand for PDCDC1	(1)
PDCDC1	Programmed cell death protein 1 (PD-1) and is the receptor for PDL1	(1)
CTLA	Cytotoxic T-lymphocyte-associated protein 4	(4)
LAG3	Lymphocyte-activation gene 3	(21)
TIM3	T-cell immunoglobulin and mucin-domain containing-3 (TIM-3) also known as HAVCR2	(22)
TIGIT	T-cell immunoreceptor with Ig and ITIM domains (WUCAM or VSTM3)	(23)
ICOS	Inducible T-cell costimulatory protein	(24)
IDO1	Indoleamine-pyrrole 2,3-dioxygenase (INDO or IDO1)	(25)
BTLA	B- and T-lymphocyte attenuator	(26,27)

### *Single cell RNA-seq, in silico* microenvironment quantification and molecular subtyping

CRC cell type specific gene expression of *IL2RB* was assessed in 363 molecular profiles from 11 CRC patients using publically available Illumina HiSeq 2000 single cell RNA-seq dataset GSE81861 (mast cell profile excluded, *n* = 1). Publically available Illumina NextSeq 500 MC38 murine single CD8+ T cell RNA-seq (*n* = 1192) from colon tumors were accessed from dataset GSE120909. FPKM expression values were downloaded from Gene Expression Omnibus (GEO) and log2 transformed with a +1 pseudo count (log2FPKM+1) and the threshold for *IL2RB* mRNA expression was determined as ≥1 log2FPKM+1. The immune and stromal microenvironment was quantified from CRC TCGA data at the transcript level by employing the microenvironment cell population (MCP) counter R package; correlation analysis was performed using the corrplot and hmsic R packages (28). Consensus Molecular Subtyping (CMS) and Colorectal Cancer Intrinsic Subtyping (CRIS) were performed on the gene expression data as previously published, using the random forest classifier and nearest template prediction model respectively (29–31).

### Gene expression patient cohorts

Gene expression and clinical data for the CRC TCGA dataset was extracted from www.cbioportal.org. Both RNA-seq and Agilent microarray data (probes collapsed to median expression) were used for gene signature analysis. The RNA-seq pipeline (RSEM log2 normalized) was matched to clinical data for disease-free survival (DFS) analysis (*n* = 322). The publically available gene expression datasets GSE39582 and GSE103479 were downloaded from GEO. Patient-matched CD3 and CD8 immunohistochemistry scores were provided for GSE103479 dataset by author DL as previously published (32).

### Immunohistochemistry and digital pathology assessment

A suitable IL2RB IHC antibody was identified, based upon assessed expression across six tumor types including CRC from the Human Protein Atlas (https://www.proteinatlas.org/). Expression of IL2RB was subsequently evaluated in an established in-house colon cancer TMA using immunohistochemistry and image analysis. TMA construction and clinicopathological characteristics of the stage II/III colon cancer patients (*n* = 631) are described elsewhere (33); however, in this study we only assessed MSI-H tumors (*n* = 115). Microsatellite instability (MSI) status was assessed by PCR using the Promega Microsatellite Instability Status kit of genetic material from the pathology specimens from which the TMA was generated. The rationale for assessing only MSI-H tumors relates to the fact that response to immune checkpoint blockade is almost exclusively observed in this subtype of patients. We hypothesise that both intrinsic cancer cell immunogenicity (through MSI-H status) and tumor microenvironment (as measured by IL2RB expression) are required for predicting response immune checkpoint blockade. Immunohistochemistry was performed for IL2RB (Polyclonal Anti-IL2RB Antibody; Atlas Antibodies, Voltavägen, Sweden; catalogue number: HPA062657; 1:1000 dilution; 15 min incubation at room temperature) on the Leica BOND-MAX automated immunostainer (Antigen retrieval: ER2 for 20 min; Detection chemistry: Bond Polymer Refine Detection and Enhancer). The optimized protocols for CD3, CD4, CD8, FOXP3, ICOS and PDL1 IHC antibodies are included in Supplementary Table S1. Slides were scanned using an Aperio AT2 at 40× magnification. IL2RB expression for each patient was calculated as the average number of IL2RB positive cells per mm^2^ across replicate cores using open-source software QuPath version 0.1.2 (34). All tissue samples from the Belfast and the South Eastern Health and Social Care Trust (HSCT) were obtained under the auspices of the Northern Ireland Biobank (www.nibiobank.org), which has ethical approval (ref: 11/NI/0013) to collect, store and distribute samples to researchers. The present study has ethical approval from NIB (reference. NIB15–0168)

### Statistical analysis

Patients with a DFS of zero months were excluded from both discovery and validation cohorts. No further filtering was performed based upon clinical pathological parameters for either cohort (e.g., all stages, all treatment groups were included). Missing-indicator method was used to account for missing clinical data in the patient cohorts for univariate and multivariate survival analysis and forest plots were performed using survivalAnalysis package in R version 3.3.1 (2016–06–21) – ‘Bug in Your Hair’. All parameters that were statistically significant by univariate survival analysis and clinically relevant were taken forward for multivariate analysis. Kaplan–Meier curves were generated using GraphPad Prism 6. The significance threshold was set at (*P* < 0.05) for all statistical tests unless stated otherwise. Welch’s *T*-test was used to determine the difference between two groups of unequal variance, Mann–Whitney test for nonparametric testing and analysis of variance (ANOVA) for comparing more than two groups. The significance of the relationship between categorical variables was determined using the Chi-squared test in R.

## RESULTS

### ACE identifies *IL2RB* as associated with immune-checkpoint expression in CRC

**Figure 1.**
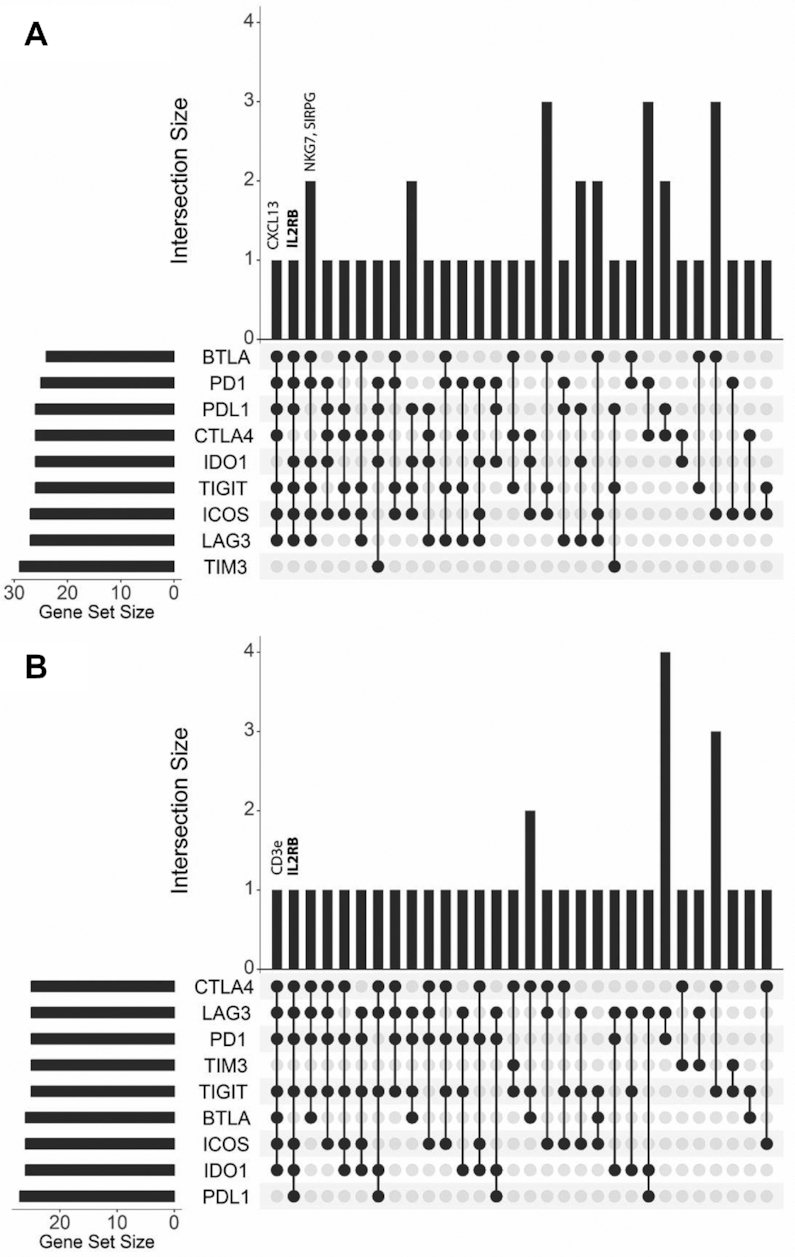
Evolutionary genetic algorithm based tool (ACE) highlights *IL2RB* as the most common intersection between analyses of nine immune checkpoint markers in CRC, across both TCGA mRNA microarray and RNA-seq datasets. (**A** and **B**) UpSet plot showing the number of intersections produced by ACE gene lists for each biomarker in both matched microarray data and RNA-seq data, respectively.

We performed ACE analysis on nine immune-checkpoints (*PD-1, CTLA4, LAG3, TIM-3, TIGIT, BTLA, ICOS, IDO1* and *PDL1*) in the CRC TCGA RNA-seq and microarray pipelines. Commonality or intersections across the nine analyses were visualized using UpSet plots and genes were reported if they were found to overlap in ≥6 lists (Figure 1A and B). The four common genes observed from the ACE analyses of the microarray pipeline were *IL2RB, CXCL13, NKG7* and *SIRPG* and the two common genes from the RNA-seq analyses were *IL2RB* and *CD3E*. *IL2RB* was identified as the most common intersection, appearing in both the microarray and RNA-seq ACE analyses of immune-checkpoints and so was taken forward for further investigation (see supplementary data for all raw exported ACE analyses).

### Clinical and pathological associations of *IL2RB*

**Figure 2.**
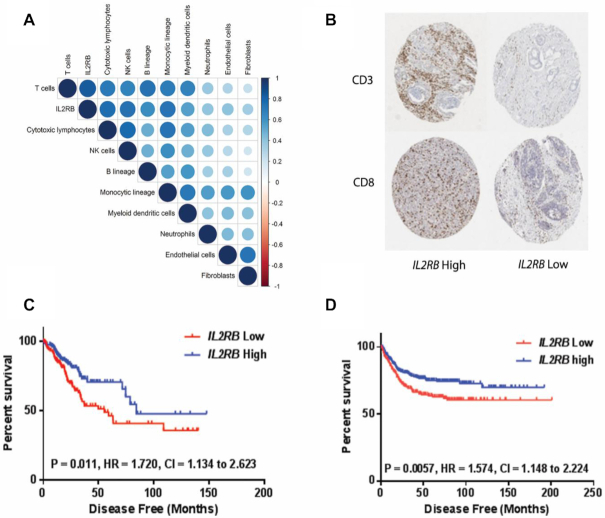
Transcriptional quantification of microenvironment and prognostic value of *IL2RB* mRNA expression in CRC. (**A**) Correlation of *IL2RB* expression with microenvironment cell population (MCP) scores for individual cell types in TCGA CRC microarray data. (**B**) Representative CD3 and CD8 IHC images generated from an in-house TMA split by median *IL2RB* mRNA expression using matched transcriptional profiles from GSE103479 (×5 magnification). (**C**) Kaplan–Meier curve showing improved DFS for patients with higher expression of *IL2RB* in CRC TCGA RNA-seq dataset (*n* = 322, log rank *P* = 0.011). (**D**) Kaplan–Meier curve showing improved DFS for patients with higher expression of *IL2RB* in GSE39582 (*n* = 519, log rank *P* value = 0.0057).

Given that *IL2RB* signaling is associated with the expansion of immune cells (17,18), we quantified the microenvironment cell populations in the TCGA CRC cohort using MCP counter, correlating each population with the expression of *IL2RB*. We observed that *IL2RB* has a strong positive correlation with an increased abundance of cytotoxic lymphocytes, T-cells, NK cells and B-cells and a weak positive correlation with fibroblasts and endothelial cells (Figure 2A). Next, we assessed the relationship between *IL2RB* mRNA expression and patient-matched CD8 and CD3 IHC expression in stage II/III CRC dataset GSE103479. We performed a Welch’s *T*-test between *IL2RB* low and high groups (median split) in stromal regions (SR), invasive front (IF) and tumor body (TB) for CD3 and CD8 IHC-positive cells. We observed significantly more CD3-positive immune cells (*P* = 0.01) in the SR of patients with high *IL2RB* compared to low, and a trend toward significance in the TB region with CD3 expression. Although not significant, CD8 expression trended toward being higher in the *IL2RB* high tumor body group (Supplementary Figure S1). Representative IHC images for CD3 and CD8 (×5 magnification) were identified from the upper and lower quintile of expression in the TB for both CD3 and CD8 (Figure 2B). Our results indicate that *IL2RB* expression may be associated with immune infiltrates, indicative of good prognosis. On this basis, we performed survival analysis to assess the prognostic value of *IL2RB* expression in the CRC TCGA RNA-seq patient cohort. Using a previously published method (35), we determined the optimal split into high and low *IL2RB* expression, based upon DFS, to be the 43rd percentile. Kaplan–Meier survival analysis demonstrated that patients in the high IL2RB subgroup had improved DFS compared to patients in the *low* IL2RB group. (Figure 2C, *n* = 322, log-rank *P* value = 0.011). We validated the prognostic value of *IL2RB* in the large publically available all-stage CRC cohort GSE39582, using the same 43rd percentile split. In this analysis, we also observed that the high *IL2RB* expressing group had improved DFS compared to the low expressing group (Figures 2D and 4A, *n* = 519, logrank *P* value = 0.006). Using Chi-squared analysis, we demonstrated that patients in the *IL2RB* high expressing group are associated with dMMR, BRAF mutations, CIMP positivity, CIN negativity, CMS1 and CRIS-B subtypes (Supplementary Table S1). Importantly, we observed using multivariate analysis in GSE39582 that *IL2RB* was an independent prognostic factor (Supplementary Figure S2, *P* < 0.01), when compared to other clinically and statistically relevant parameters. We also demonstrate in the TCGA colon cohort that IL2RB mRNA expression is significantly associated with MSI-H patients (Supplementary Figure S3, *P* < 0.0001).

### *IL2RB* single cell RNA-seq and IHC

**Figure 3.**
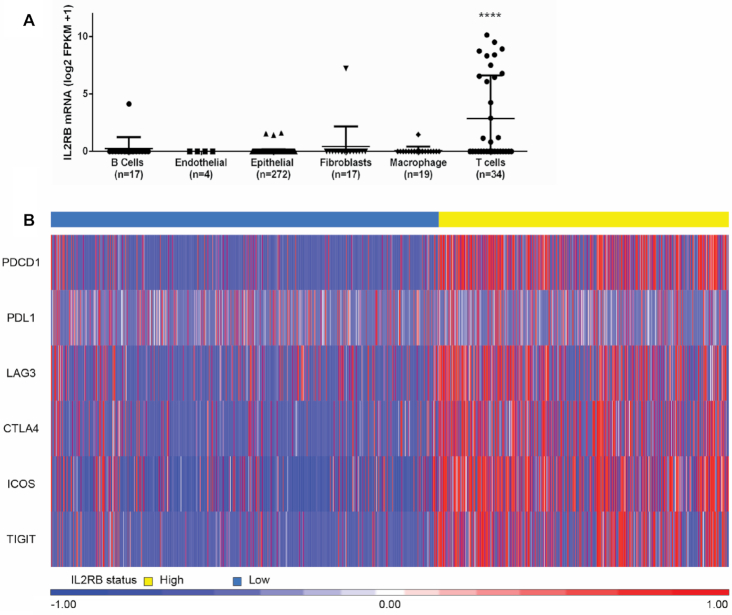
Single-Cell RNA-seq characterisation of *IL2RB* in CRC. (**A**) Dot plot showing expression of *IL2RB* (log2 FPKM +1) in publically available (GSE81861) single cell RNA-seq profiles from 11 CRC patients compared across cell type (ANOVA,*P* < 0.0001 (****)). (**B**) Heatmap comparing log2 FPKM+1 expression of *PDCD1, PDL1, LAG3, CTLA4, ICOS* and *TIGIT* in *IL2RB+* CD8+ T cells (*n* = 1192) derived from single cell MC38 Colon cancer anti-PD1 and anti-GITR treated mouse models RNA-seq dataset (GSE120909).

**Figure 4.**
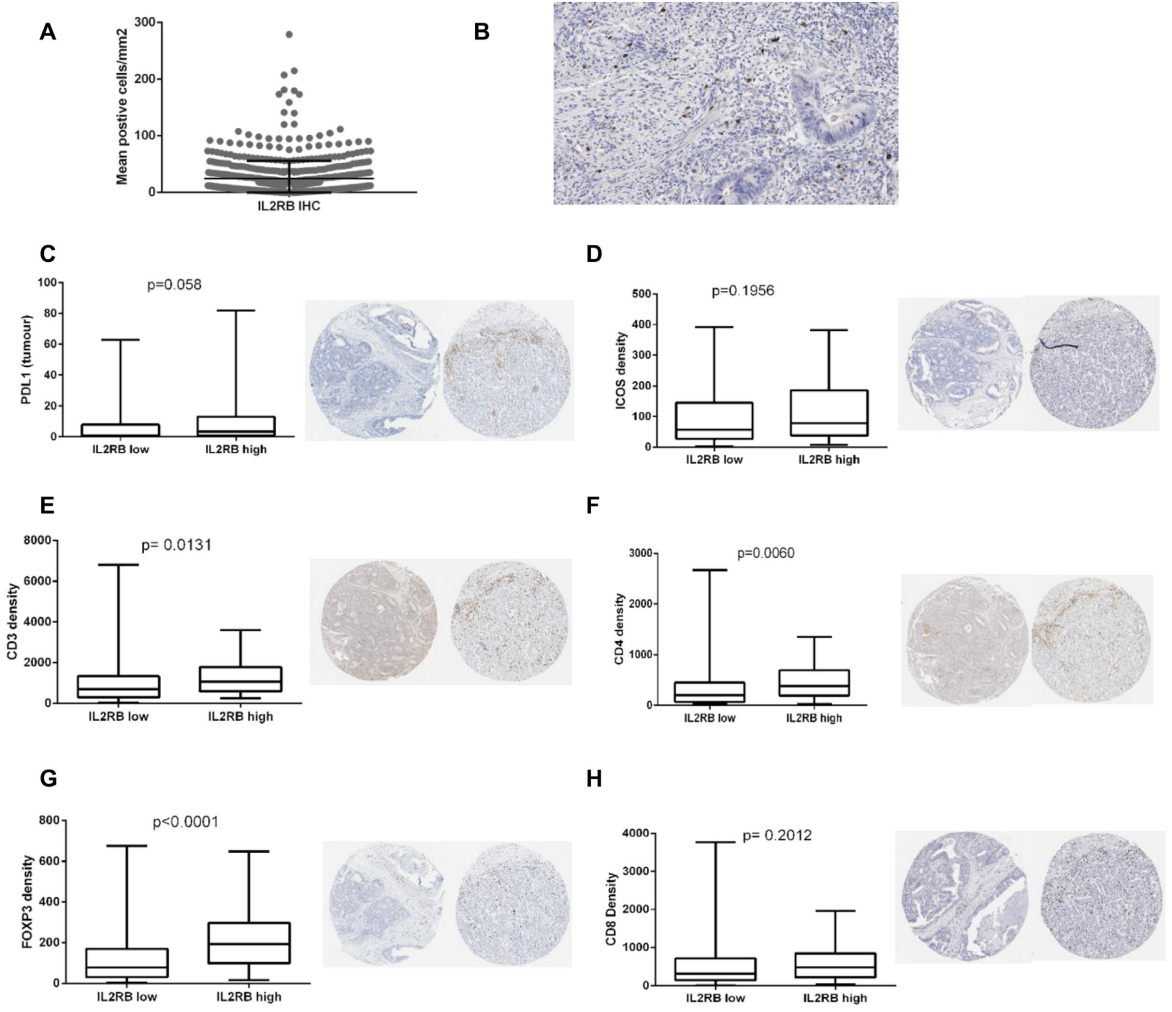
Comprehensive comparative assessment of IL2RB IHC and immune markers in stage II/III MSI-CRC (*n* = 115) from an in-house TMA. (**A**) Dot plot showing mean IL2RB positive cells/mm2. (**B**) Representative image of IL2RB IHC (×20 magnification). (**C**) Boxplot and representative images (×5 magnification) comparing PDL1+ tumor cells by IHC in IL2RB high and low patients (*P* = 0.058). (**D**) Boxplot and representative images (×5 magnification) comparing density of ICOS+ cells by IHC in IL2RB high and low patients (*P* = 0.1956). (**E**) Boxplot and representative images (×5 magnification) comparing density of CD3+ positive cells by IHC in IL2RB high and low patients (*P* = 0.0103). (**F**) Boxplot and representative images (×5 magnification) comparing density of CD4+ positive cells by IHC in IL2RB high and low patients (*P* = 0.006). (**G**) Boxplot and representative images (×5 magnification) comparing density of FOXP3+ positive cells by IHC in IL2RB high and low patients (*P*<0.0001). (**H**) Boxplot and representative images (×5 magnification) comparing density of CD8+ positive cells by IHC in IL2RB high and low patients (*P* = 0.2012). Significance determined using Mann–Whitney test. IL2RB status determined using 43rd percentile from transcriptional analysis

To delineate cell type specific expression of *IL2RB*, we utilized 363 publically available single cell RNA-seq profiles from 11 CRC patients (GSE81861). We observed that *IL2RB* was significantly upregulated in T-cells compared to all other cell types (Figure 3A, *P* < 0.0001, ANOVA). Next, we assessed whether *IL2RB* expression is associated with increased immune-checkpoint expression using single CD8+ T-cell RNA-seq data (*n* = 1192) from MC38 colon cancer mouse models, treated with immune checkpoint therapy GSE120909. With the exception of *PDL1*, we observed that *IL2RB* positive CD8+ T-cells have significantly higher immune-checkpoint expression (Figure 3B, *P* < 0.0001, Mann–Whitney test). Using the Human Protein Atlas, we identified an IL2RB IHC antibody which stains small populations of immune cells in many tumor types including CRC, melanoma, breast, lung, pancreatic and head and neck (Supplementary Figure S4). We optimized this antibody in-house and assessed the average IL2RB IHC expression per mm^2^ in a large cohort of stage II/III MSI-H colon cancer (*n* = 115). We optimized digital assessment of IL2RB IHC using the open source image analysis software QuPath and detected an average of 22 positive cells/mm^2^ (Figure 4A and C). We observed sensitive and specific staining of IL2RB protein expression on a small population of lymphocytes (Figure 4B). Next, we compared IL2RB IHC expression to a repertoire of immune markers (CD3, CD4, CD8 and FOXP3), PDL1 and ICOS expression by IHC. Using the same 43rd percentile split established for *IL2RB* expression in the transcriptomics analysis, we observed a trend toward increased PDL1 tumor (Figure 4C, *P* = 0.058) and ICOS expression (Figure 4D, *P* = 0.1956) in the IL2RB^Hi^ patients. We also observed a significant increase in the density of CD3 (Figure 4E, *P* = 0.0131), CD4 (Figure 4F, *P* = 0.006) and FOXP3 (Figure 4G, *P* <0.0001) immune markers in the IL2RB^Hi^h IHC group. The density of CD8 positive cells was higher in the IL2RB^Hi^ compared to the IL2RB^Lo^ patients but was not significant (Figure 4H, *P* = 0.2012).

### *IL2RB* as a potential predictive biomarker

Immune checkpoint clinical trials in dMMR CRC patients such as CheckMate 142 (anti-PD1) show objective response rates of 31.1% (39). There is therefore a need for a robust biomarker that identifies this subgroup of dMMR CRC patients that respond to immune checkpoint therapy. Therefore, we wished to assess the potential predictive value of *IL2RB* for response to immune-checkpoint therapy. Given the paucity of experimental data and gene signatures currently available for immune-checkpoint therapy in CRC, we utilized three gene signatures generated in urothelial cancer and melanoma. The durvalumab (anti-PDL1) gene signature generated in urothelial cancer was shown to have a strong positive correlation with *IL2RB* expression in both the CRC microarray and RNA-seq pipelines (Supplementary Figure S5A and B, *R* = 0.87, *P* < 0.0001 and *R* = 0.82, *P* < 0.0001) (36). The NK cell/Anti-PD-1 signature devised in melanoma models also strongly correlated with *IL2RB* (Supplementary Figure S5C and D, *R* = 0.77, *P* < 0.0001 and *R* = 0.77, *P* < 0.0001) as did the pembrolizumab signature from the KEYNOTE-001 phase I clinical trial (Supplementary Figure S5E and F, *R* = 0.88, *P* < 0.0001 and *R* = 0.84, *P* < 0.0001) (37,38). Our observations provide positive evidence that *lL2RB* is significantly associated with response to immune checkpoint therapies.

## DISCUSSION

A subset of MSI-H/dMMR mCRC patients experience durable response to immune-checkpoint therapies. Results from trials such as CheckMate-142 (NCT02060188) indicate that anti-PD1 and anti-CTLA4 therapies could become first-line treatment for this patient group (39). Moreover, exciting preliminary results from phase II clinical trial NICHE (NCT03026140) for early stage dMMR colon cancer suggest that a larger proportion of dMMR/MSI-H stage II/III CRC patients may benefit from immune-checkpoint therapy than in the metastatic setting (40). Aside from dMMR/MSI-H, one of the most widely used predictive biomarkers for anti-PD1 therapy is PDL1 IHC; however, conflicting results from studies regarding definitive cut-off thresholds, tumor/ stromal staining and poor inter-reader concordance results mean that it is not routinely used for CRC, thus highlighting that a more robust predictive biomarker approach is required. Similarly, biomarker-based stratification beyond MSI-H/dMMR for other immune-checkpoint therapies is lacking and requires further investigation (41,42).

In this study, we employed our previously-published platform ACE to extract genes correlated with the expression of immune-checkpoints currently under investigation or being employed as therapeutic targets in clinical studies in CRC. ACE utilizes an evolutionary genetic algorithm rather than classical correlation analysis. It is an alternative form of feature selection which has the potential to assess a much larger combination of correlates across subsets of features and while performing random sampling of the observations. We hypothesized that commonality across our ACE analysis of nine selected immune-checkpoints may reveal a novel overarching predictive biomarker for certain immune-checkpoint based therapies. Our analyses of both microarray and RNA-seq TCGA CRC gene expression data revealed *IL2RB* to be the most common co-expressed gene (intersection), featuring in 6/9 of immune-checkpoint gene lists generated by ACE.

*IL2RB* is part of a receptor signaling complex that also consists of alpha and gamma receptor subunits and its functions are highly pleiotropic (43). *IL2RB* activation via endogenous IL2 or biased therapeutic stimulation results in the expansion of anti-tumor immune cells, in particular CD8+, CD4+ and NK cells. IL2RB was recently shown to be significantly upregulated in CRC, specifically in cytolytic-high tumors. Additionally, the Treg marker FOXP3 were also significantly higher in cytolytic-high CRCs. In contrast, a number of studies have demonstrated that IL2RB positive immune cells are associated with immune suppression and T-cell exhaustion. However, their exact function within the context of immune checkpoint therapy remains unclear (16,45–51). The IL2RB-biased engineered cytokine NKTR-214 significantly increases the ratio of CD8 CTLs to immunosuppressive CD4 FOXP3 T-regulatory cells, creating a potent anti-tumor environment, while also increasing the expression of immune-checkpoints such as CD274 (PDL1) (18). The ‘molecular stalemate’ produced by IL2RB stimulation is therapeutically targetable and on this basis. NKTR-214 is currently under investigation in combination with anti-PD1 therapy across a range of solid tumors, with a phase III trial ongoing in advanced melanoma (NCT03635983). Given the recent resurgence of IL2-based therapies highlighted by Garber *et al.*, we decided to comprehensively investigate IL2RB expression within the context of CRC and immune-checkpoints (19).

First, we demonstrated using transcriptional analysis that *IL2RB* mRNA expression is associated with increased infiltration of immune cells such as T cells and cytotoxic lymphocytes, which are known to be associated with improved outcomes in CRC patients and the good-prognosis immune CMS1 molecular subtype. Further support for a key role for IL2RB is provided by our survival analysis of the CRC TCGA cohort, where patients with higher *IL2RB* gene expression had improved DFS versus those with lower expression levels. We validated this observation in a large independent cohort and demonstrated that higher *IL2RB* gene expression was an independent prognostic factor by multivariate survival analysis. It is well-established that tumors with increased TILs have improved outcomes and that a primed immune infiltrate is a prerequisite for immune-checkpoint therapy response. To delineate the cellular origin of *IL2RB* expression, we interrogated publically available single-cell RNA-seq profiles from the CRC tumor and microenvironment of 11 patients and determined that *IL2RB* is predominantly expressed on a subset of T-cells. The exact function of IL2RB-expressing T cells are yet to be fully elucidated.

T cells are widely known to be one of the main effector populations of immune-checkpoint therapies, it has been reported that IL2RB expression on CD8+ T cells may play a role in exhaustion in a variety of contexts including viral infection and thus this role should also be investigated within the context of cancer (16,45–53). Our analysis of single-cell RNA-seq profiles (*n* = 1192) from the publically available dataset GSE120909 showed that *IL2RB*+ CD8 T-cells in MC38 murine colon cancer models were unequivocally associated with immune-checkpoint expression. To visualize the specific cells expressing IL2RB, we performed IL2RB IHC on a TMA from large cohort MSI-H colon cancer patients (*n* = 115). IHC digital assessment indicated that *IL2RB* is expressed on a small population of TILs. Next, we demonstrated that MSI-H CRC patients with increased IL2RB+ immune cells have an increased abundance of CD3, CD4 and FOXP3 TILs and higher PDL1 tumor and ICOS expression. Interestingly, CD8 immune cells were not significantly altered in the IL2RB IHC high patients. This supports the recent observations that IL2RB expression is correlated with mRNA expression of FOXP3 in cytolytic (CYT-high) colorectal tumors (44). We hypothesize that these *IL2RB* -positive cells represent an important and distinct subset of immune cells that may influence immune-checkpoint regulation and an exhausted yet targetable T-cell phenotype in CRC.

Having established that *IL2RB+* T-cells in CRC are associated with immune-checkpoint expression, we investigated whether *IL2RB* may have utility as a predictive biomarker for CRC patients receiving immune-checkpoint blockade therapy. A limitation to this study is the lack of CRC-specific data currently available for biomarker-informed evaluation of response to immune checkpoints. As a consequence of the dearth of CRC-specific trial gene expression data available for this study, we employed three previously published predictive gene signatures for anti-PD1 and anti-PDL1 therapies, which had been generated in melanoma, lung and urothelial cancer cohorts (36–38). We observed strong positive correlation of *IL2RB* with predictive gene signatures for pembrolizumab and durvalumab, across both microarray and RNA-seq in the CRC TCGA cohorts. Our results from previously published predictive gene signatures generated in immune hot tumors such as melanoma, lung and urothelial cancers indicate that *IL2RB* has a strong association with the biology that underpins response to immune-checkpoint therapy and could be extrapolated to immune ‘hot’ MSI-H CRC tumors. We therefore hypothesize that IL2RB may have predictive value for patients receiving immune checkpoint therapy.

In conclusion, we demonstrate how our platform ACE which utilizes an evolutionary genetic algorithm can be integrated within a biomarker discovery pipeline. Using ACE, we have highlighted *IL2RB* expression is unequivocally linked with immune-checkpoint genes in CRC. We believe *IL2RB* may represent an important player in the immune landscape of CRC and should continue to be investigated as a predictive biomarker with potential clinical utility for CRC patients receiving immune-checkpoint blockade.

## Supplementary Material

lqab016_Supplemental_Files
