# Supplementary material for: Evolutionary genetic algorithm identifies IL2RB as a potential predictive biomarker for immune-checkpoint therapy in colorectal cancer
Source: NAR Genom Bioinform. 2021 Apr 20;3(2):lqab016. doi: 10.1093/nargab/lqab016 (PMC8057496; doi:10.1093/nargab/lqab016)
Supplement: lqab016_Supplemental_Files [file lqab016_supplemental_files.zip › Supplementary_Tables_&_Figures_revised.pptx]

## Slide 1
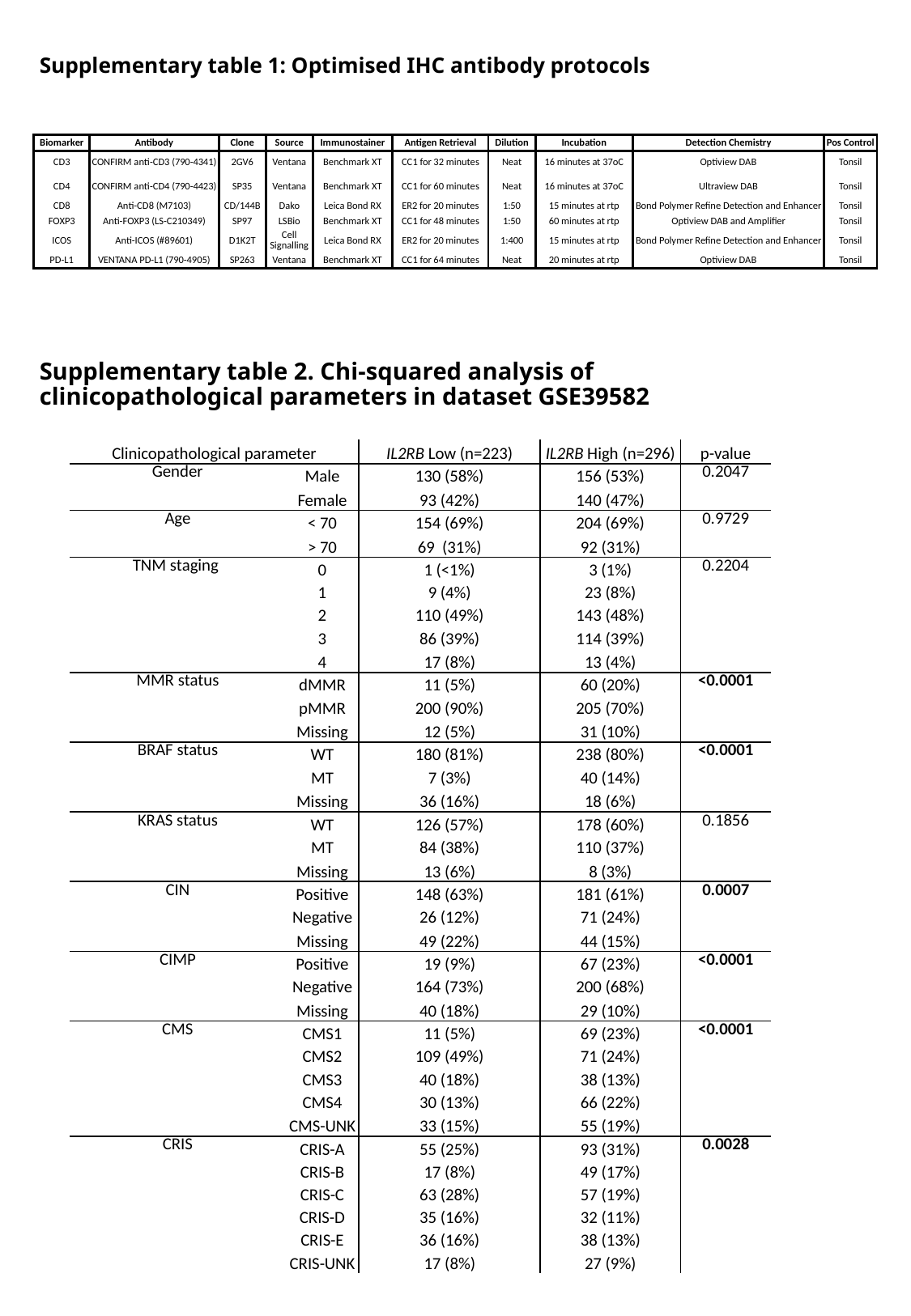

Supplementary table 1: Optimised IHC antibody protocols
| Biomarker | Antibody | Clone | Source | Immunostainer | Antigen Retrieval | Dilution | Incubation | Detection Chemistry | Pos Control |
| --- | --- | --- | --- | --- | --- | --- | --- | --- | --- |
| CD3 | CONFIRM anti-CD3 (790-4341) | 2GV6 | Ventana | Benchmark XT | CC1 for 32 minutes | Neat | 16 minutes at 37oC | Optiview DAB | Tonsil |
| CD4 | CONFIRM anti-CD4 (790-4423) | SP35 | Ventana | Benchmark XT | CC1 for 60 minutes | Neat | 16 minutes at 37oC | Ultraview DAB | Tonsil |
| CD8 | Anti-CD8 (M7103) | CD/144B | Dako | Leica Bond RX | ER2 for 20 minutes | 1:50 | 15 minutes at rtp | Bond Polymer Refine Detection and Enhancer | Tonsil |
| FOXP3 | Anti-FOXP3 (LS-C210349) | SP97 | LSBio | Benchmark XT | CC1 for 48 minutes | 1:50 | 60 minutes at rtp | Optiview DAB and Amplifier | Tonsil |
| ICOS | Anti-ICOS (#89601) | D1K2T | Cell Signalling | Leica Bond RX | ER2 for 20 minutes | 1:400 | 15 minutes at rtp | Bond Polymer Refine Detection and Enhancer | Tonsil |
| PD-L1 | VENTANA PD-L1 (790-4905) | SP263 | Ventana | Benchmark XT | CC1 for 64 minutes | Neat | 20 minutes at rtp | Optiview DAB | Tonsil |
# Supplementary table 2. Chi-squared analysis of clinicopathological parameters in dataset GSE39582
| Clinicopathological parameter | | IL2RB Low (n=223) | IL2RB High (n=296) | p-value |
| --- | --- | --- | --- | --- |
| Gender | Male | 130 (58%) | 156 (53%) | 0.2047 |
| | Female | 93 (42%) | 140 (47%) | |
| Age | < 70 | 154 (69%) | 204 (69%) | 0.9729 |
| | > 70 | 69 (31%) | 92 (31%) | |
| TNM staging | 0 | 1 (<1%) | 3 (1%) | 0.2204 |
| | 1 | 9 (4%) | 23 (8%) | |
| | 2 | 110 (49%) | 143 (48%) | |
| | 3 | 86 (39%) | 114 (39%) | |
| | 4 | 17 (8%) | 13 (4%) | |
| MMR status | dMMR | 11 (5%) | 60 (20%) | <0.0001 |
| | pMMR | 200 (90%) | 205 (70%) | |
| | Missing | 12 (5%) | 31 (10%) | |
| BRAF status | WT | 180 (81%) | 238 (80%) | <0.0001 |
| | MT | 7 (3%) | 40 (14%) | |
| | Missing | 36 (16%) | 18 (6%) | |
| KRAS status | WT | 126 (57%) | 178 (60%) | 0.1856 |
| | MT | 84 (38%) | 110 (37%) | |
| | Missing | 13 (6%) | 8 (3%) | |
| CIN | Positive | 148 (63%) | 181 (61%) | 0.0007 |
| | Negative | 26 (12%) | 71 (24%) | |
| | Missing | 49 (22%) | 44 (15%) | |
| CIMP | Positive | 19 (9%) | 67 (23%) | <0.0001 |
| | Negative | 164 (73%) | 200 (68%) | |
| | Missing | 40 (18%) | 29 (10%) | |
| CMS | CMS1 | 11 (5%) | 69 (23%) | <0.0001 |
| | CMS2 | 109 (49%) | 71 (24%) | |
| | CMS3 | 40 (18%) | 38 (13%) | |
| | CMS4 | 30 (13%) | 66 (22%) | |
| | CMS-UNK | 33 (15%) | 55 (19%) | |
| CRIS | CRIS-A | 55 (25%) | 93 (31%) | 0.0028 |
| | CRIS-B | 17 (8%) | 49 (17%) | |
| | CRIS-C | 63 (28%) | 57 (19%) | |
| | CRIS-D | 35 (16%) | 32 (11%) | |
| | CRIS-E | 36 (16%) | 38 (13%) | |
| | CRIS-UNK | 17 (8%) | 27 (9%) | |

## Slide 2
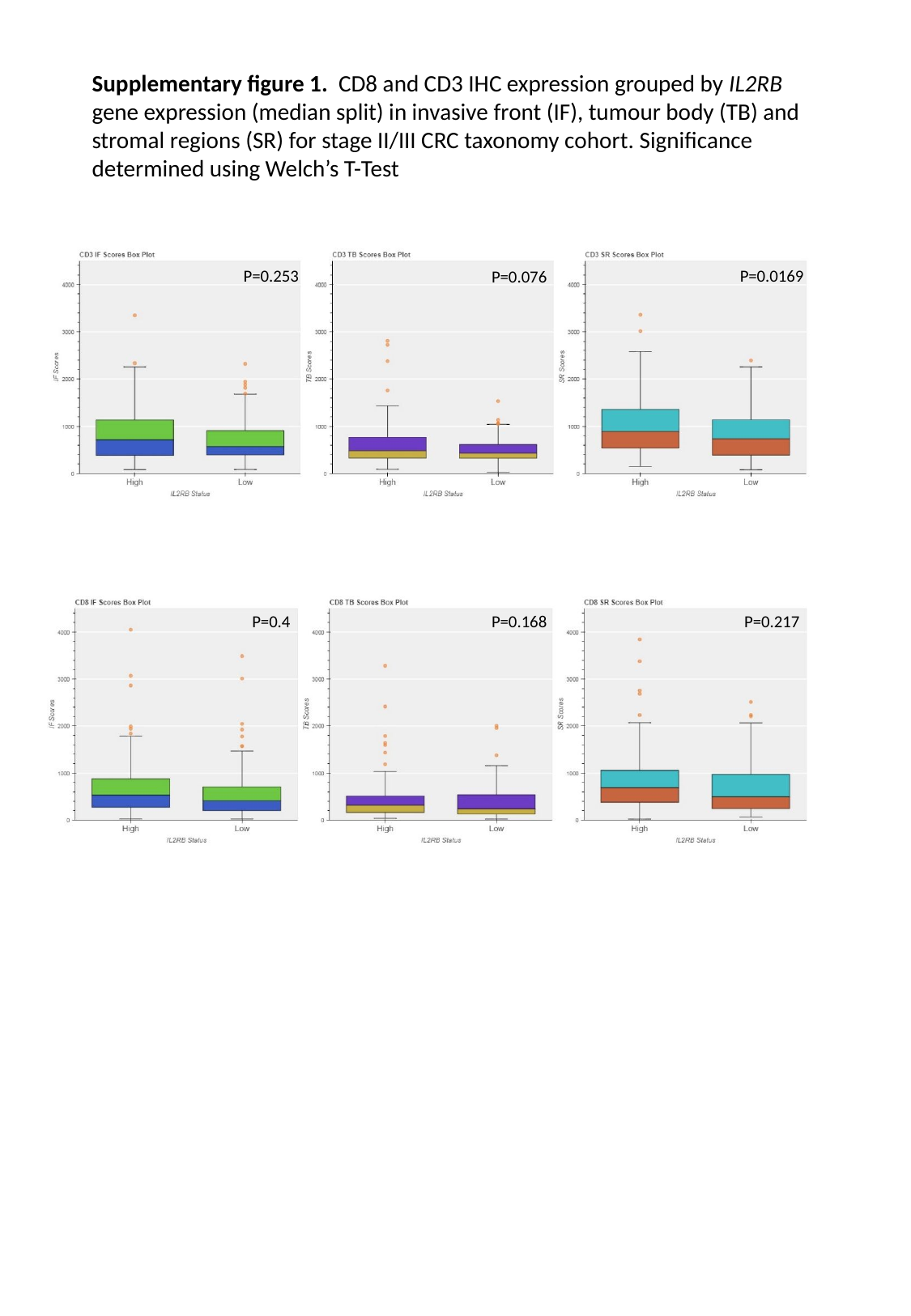

Supplementary figure 1. CD8 and CD3 IHC expression grouped by IL2RB gene expression (median split) in invasive front (IF), tumour body (TB) and stromal regions (SR) for stage II/III CRC taxonomy cohort. Significance determined using Welch’s T-Test
P=0.253
P=0.0169
P=0.076
P=0.217
P=0.168
P=0.4

## Slide 3
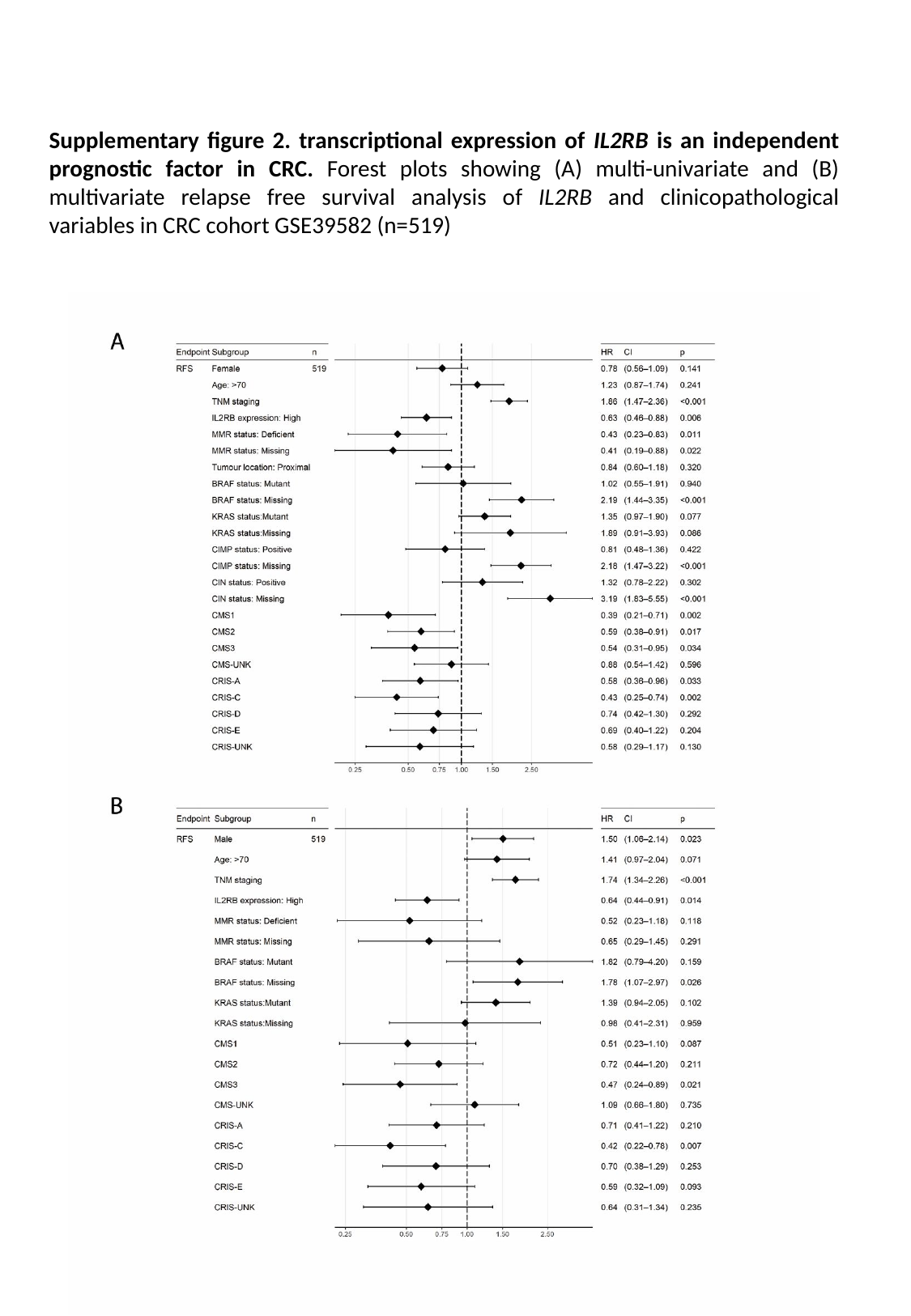

Supplementary figure 2. transcriptional expression of IL2RB is an independent prognostic factor in CRC. Forest plots showing (A) multi-univariate and (B) multivariate relapse free survival analysis of IL2RB and clinicopathological variables in CRC cohort GSE39582 (n=519)

## Slide 4
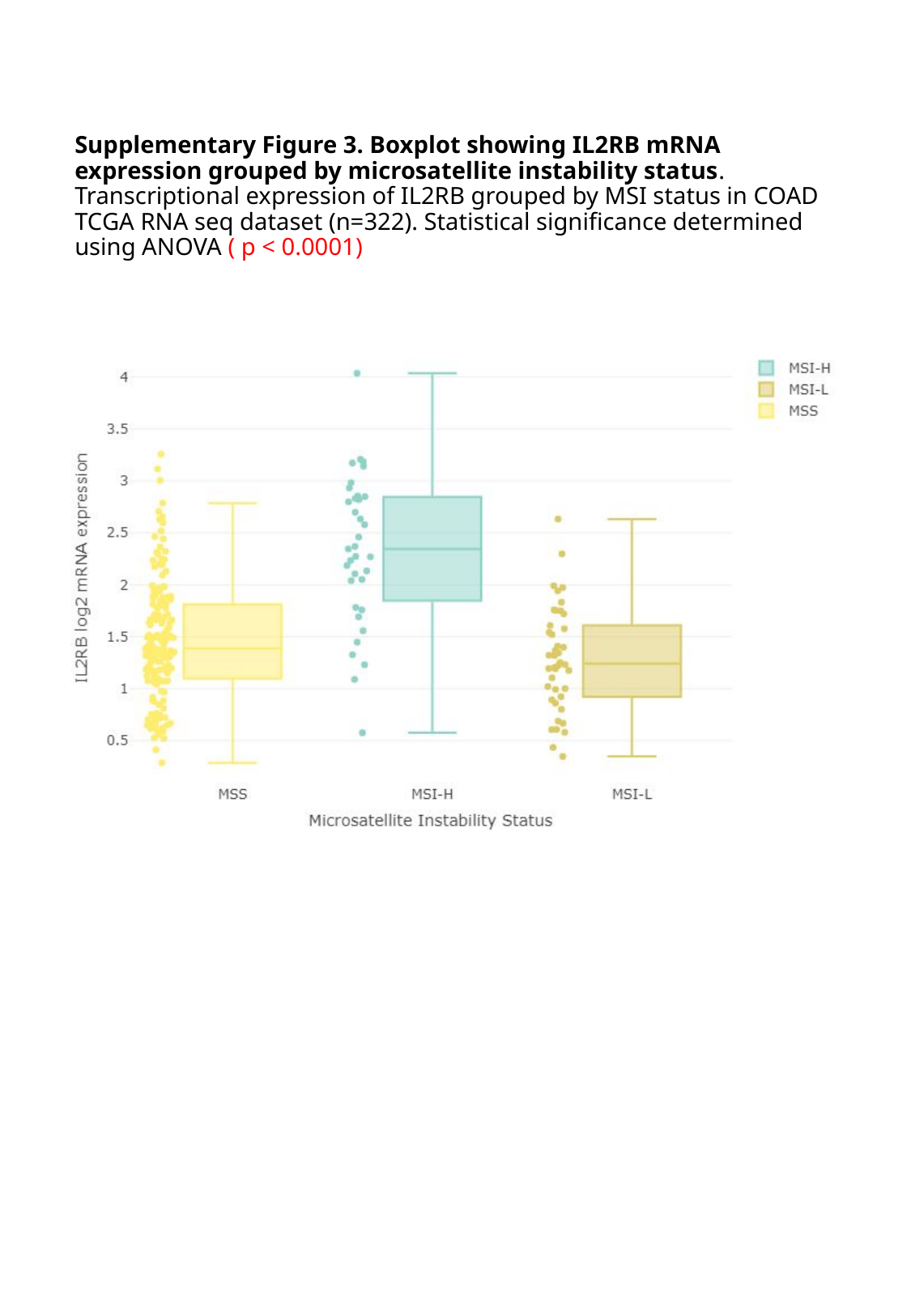

# Supplementary Figure 3. Boxplot showing IL2RB mRNA expression grouped by microsatellite instability status. Transcriptional expression of IL2RB grouped by MSI status in COAD TCGA RNA seq dataset (n=322). Statistical significance determined using ANOVA ( p < 0.0001)

## Slide 5
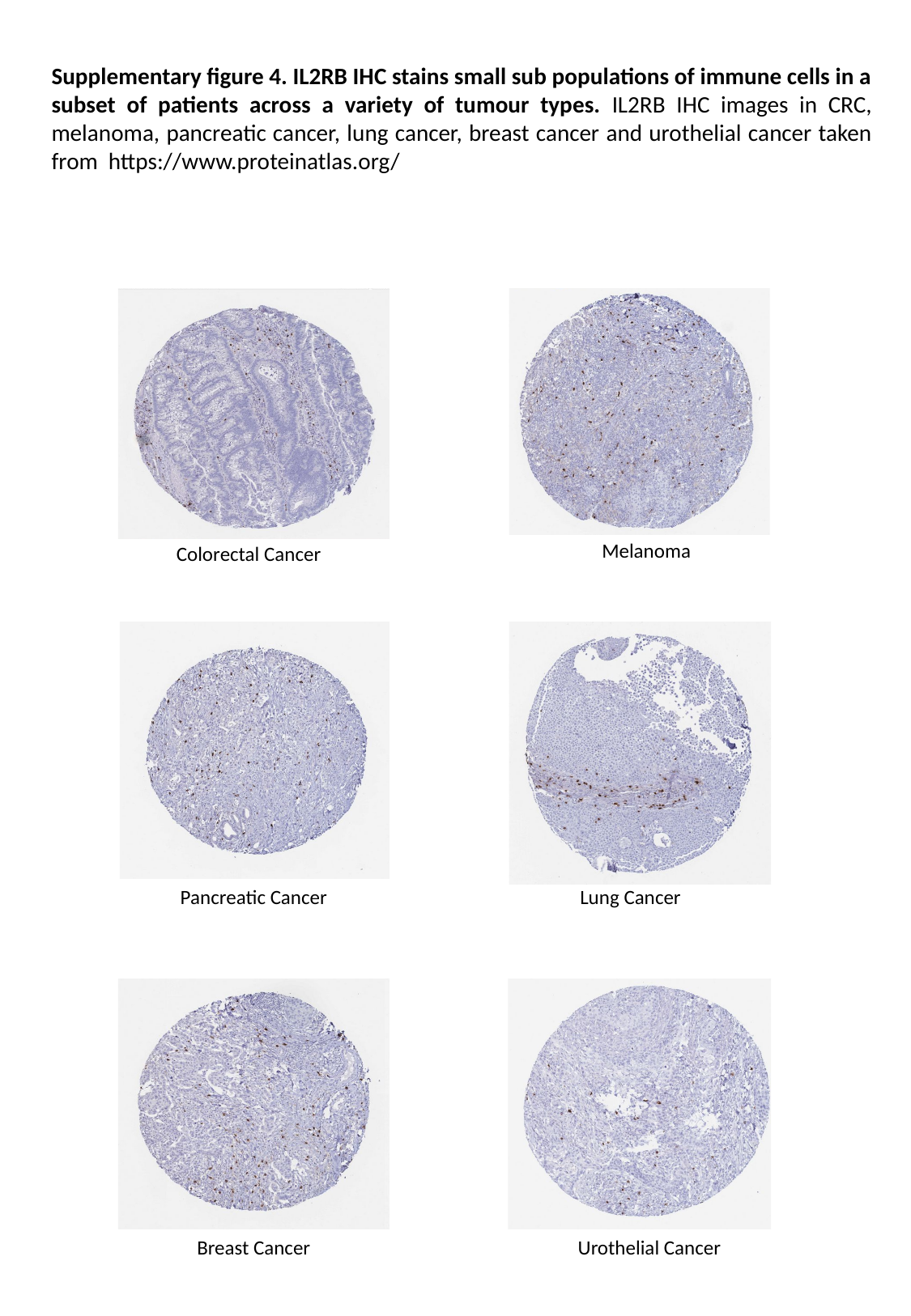

Supplementary figure 4. IL2RB IHC stains small sub populations of immune cells in a subset of patients across a variety of tumour types. IL2RB IHC images in CRC, melanoma, pancreatic cancer, lung cancer, breast cancer and urothelial cancer taken from https://www.proteinatlas.org/
Melanoma
Colorectal Cancer
Pancreatic Cancer
Lung Cancer
Breast Cancer
Urothelial Cancer

## Slide 6
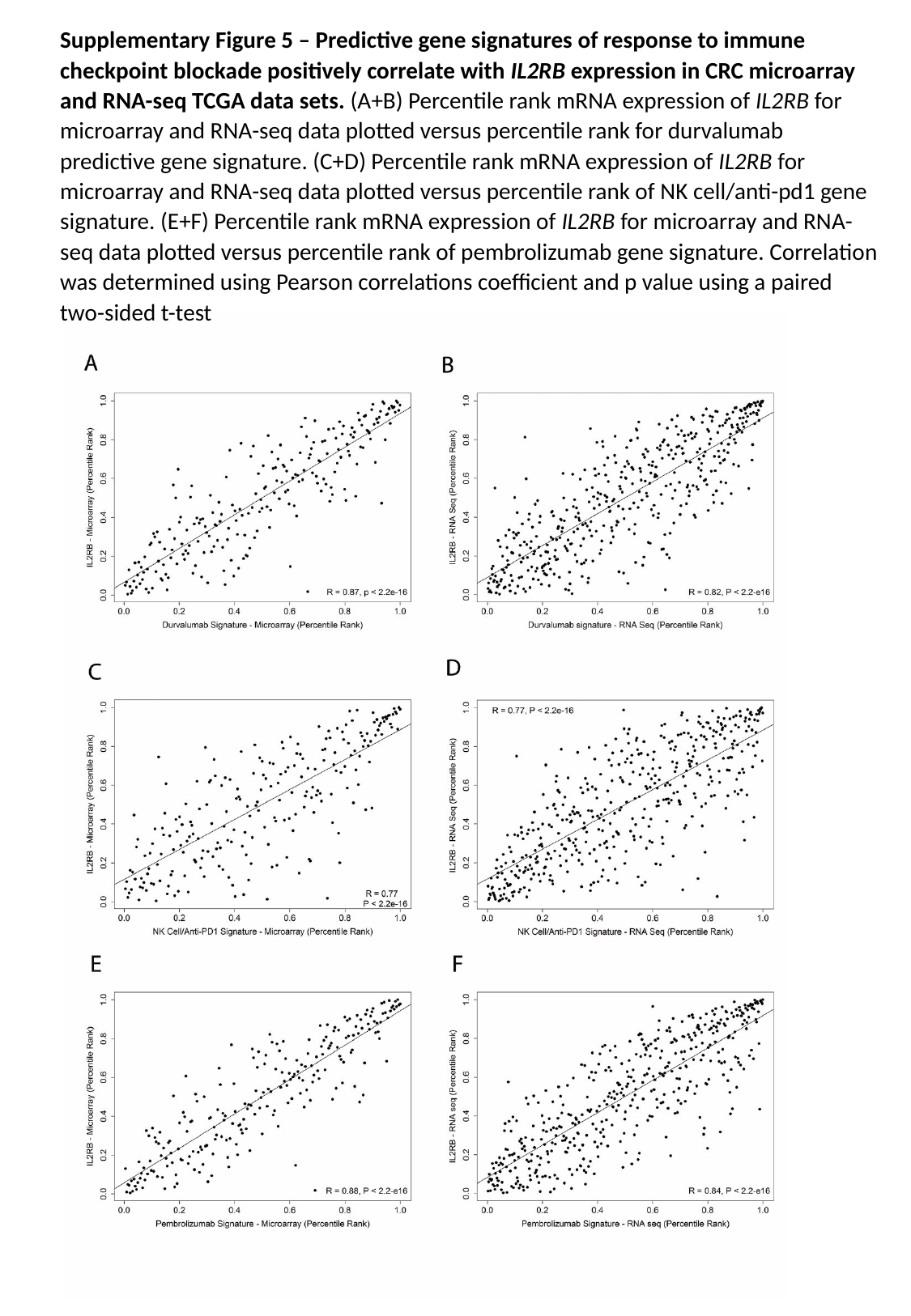

Supplementary Figure 5 – Predictive gene signatures of response to immune checkpoint blockade positively correlate with IL2RB expression in CRC microarray and RNA-seq TCGA data sets. (A+B) Percentile rank mRNA expression of IL2RB for microarray and RNA-seq data plotted versus percentile rank for durvalumab predictive gene signature. (C+D) Percentile rank mRNA expression of IL2RB for microarray and RNA-seq data plotted versus percentile rank of NK cell/anti-pd1 gene signature. (E+F) Percentile rank mRNA expression of IL2RB for microarray and RNA-seq data plotted versus percentile rank of pembrolizumab gene signature. Correlation was determined using Pearson correlations coefficient and p value using a paired two-sided t-test
